# Liposarcoma of gallbladder: a case report and literature review

**DOI:** 10.1093/jscr/rjab273

**Published:** 2021-06-22

**Authors:** Yuta Ushida, Hiromichi Ito, Yosuke Inoue, Takafumi Sato, Yoshihiro Ono, Atsushi Oba, Yu Takahashi

**Affiliations:** Division of Hepatobiliary and Pancreatic Surgery, Cancer Institute Hospital, Japanese Foundation for Cancer Research, Koto-ku, Tokyo 135-8550, Japan; Division of Hepatobiliary and Pancreatic Surgery, Cancer Institute Hospital, Japanese Foundation for Cancer Research, Koto-ku, Tokyo 135-8550, Japan; Division of Hepatobiliary and Pancreatic Surgery, Cancer Institute Hospital, Japanese Foundation for Cancer Research, Koto-ku, Tokyo 135-8550, Japan; Division of Hepatobiliary and Pancreatic Surgery, Cancer Institute Hospital, Japanese Foundation for Cancer Research, Koto-ku, Tokyo 135-8550, Japan; Division of Hepatobiliary and Pancreatic Surgery, Cancer Institute Hospital, Japanese Foundation for Cancer Research, Koto-ku, Tokyo 135-8550, Japan; Division of Hepatobiliary and Pancreatic Surgery, Cancer Institute Hospital, Japanese Foundation for Cancer Research, Koto-ku, Tokyo 135-8550, Japan; Division of Hepatobiliary and Pancreatic Surgery, Cancer Institute Hospital, Japanese Foundation for Cancer Research, Koto-ku, Tokyo 135-8550, Japan

## Abstract

A 53-year-old man with prior history of resection of liposarcoma in his leg presented with gallbladder mass. Computed tomography showed 4-cm tumor at gallbladder fundus with weak enhancement with IV contrast. Differential diagnoses included hemangioma and liposarcoma, and laparoscopic cholecystectomy was recommended. In the operating room, the tumor appeared without serosal and liver invasions and uncomplicated laparoscopic cholecystectomy was completed. Histopathological examination revealed the tumor as myxoid liposarcoma with round cells. Adjuvant chemotherapy was not given, and he was placed on imaging surveillance. At 16 months after the operation, he developed recurrence of liposarcoma in the left popliteal fossa.

## INTRODUCTION

Gallbladder sarcoma is rare and the most have been the subject for case report. There were 46 cases with primary gallbladder sarcoma reported in past two decades. Among them, liposarcoma of gallbladder is extremely rare and there were only five well documented in English literatures to our knowledge [1–4]. Here, we report a case of gallbladder liposarcoma which we recently experienced, with literature review for this rare disease.

**Figure 1:**
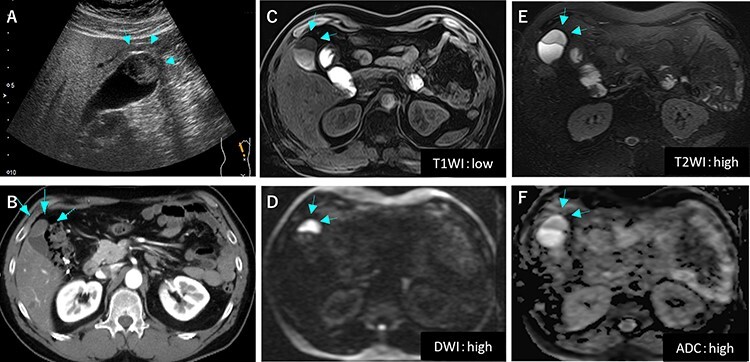
Preoperative image findings; (**A**) Gallbladder tumor was at gallbladder body by abdominal ultrasonography; (**B**) CT scan revealed slightly enhanced tumor located at gallbladder fundus; (**C**–**F**) On MRI, the tumor had high signal in both of T2 and diffusion weighed image and low signal in both of T1 and apparent diffusion coefficient.

## CASE REPORT

**Figure 2:**
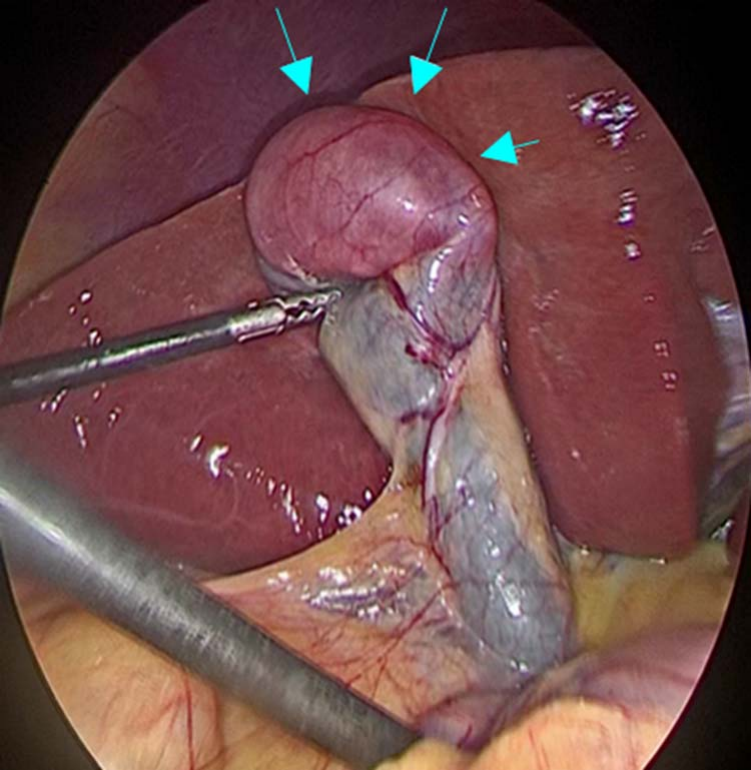
Intraoperative findings during laparoscopic cholecystectomy; The tumor was at fundus of gallbladder without serosal invasion.

**Figure 3:**
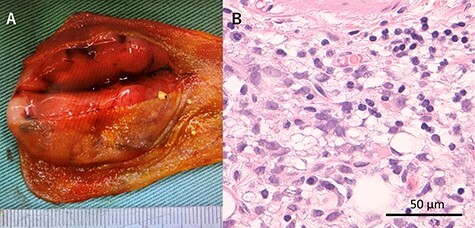
Macroscopic and microscopic findings of the resected specimen; (**A**) Tumor was solid and well-defined tumor mainly in the submucosa; (**B**) Tumor was myxoid liposarcoma consisting of solid growth of lipoblasts and round cells; There was no necrosis and proliferation of spindle and round cell component were identified in the background of myxoid stroma.

A 53-year-old man with history of liposarcoma had gallbladder tumor detected during routine ultrasonography (Fig. 1A) and was referred to us. He was first diagnosed as leg liposarcoma 5 years before and underwent complete resection. Despite of history of liposarcoma in his leg, he never developed any abdominal symptoms. Laboratory tests were unremarkable including carcinoembryonic antigen and carbohydrate antigen 19-9 (CA19-9) within their normal limits. Computed tomography (CT) scan demonstrated slightly enhanced 4-cm tumor located at gallbladder fundus (Fig. 1B). On magnetic resonance imaging (MRI), the tumor had high signal in both of T2 and diffusion weighed image and low signal in both of T1 and apparent diffusion coefficient (Fig. 1C–F). Based on these findings in imaging studies, our working differential diagnosis included liposarcoma and hemangioma rather than adenocarcinoma, and we recommended laparoscopic cholecystectomy. In the exploration, the tumor in the fundus appeared without serosal invasion (Fig. 2). Intraoperative ultrasonography revealed the tumor was contained in the gallbladder without infiltration into the liver, and in fact there was a distance between the liver bed and the tumor. Sonazoid enhanced ultrasonography ruled out liver metastasis and thus, we proceeded to laparoscopic cholecystectomy as planned. When the gallbladder was opened, yellowish white tumor was confirmed in the submucosal layer with the overlying mucosa intact (Fig. 3A). Histopathological examination revealed, this tumor as myxoid liposarcoma consisting of lipoblasts and round cells. There was no necrosis and proliferations of spindle and round cell component were identified in the background of myxoid stroma (Fig. 3B). These histological features were similar to the ones for the sarcoma in his leg that was resected before. The patient’s postoperative course was uncomplicated and was discharged home on Day 3. The adjuvant therapy was elected not to be given and he was placed on imaging surveillance with CT scan in every 3–6 months. In 16 months after this operation, he developed disease recurrence in the left popliteal fossa. There was no recurrence to date in the abdominal cavity at 28 months after the operation, he was alive with disease.

## DISCUSSION

Sarcoma of gallbladder is a rare clinical entity, and a PubMed search using key words of gallbladder and sarcoma identified 46 reported cases in past two decades [2, 4–7] (Table 1). The histopathological types of sarcoma were as shown in Table 1, and leiomyosarcoma and malignant fibrous histiocytoma variants were the most common subtype (35% for each) and liposarcoma represented for 11% of gallbladder sarcoma. Most patients underwent simple cholecystectomy and only one patient with angiosarcoma (2%) was reported to have lymph node metastasis.

**Table 1:** Reported cases of gallbladder sarcoma with resection

Characteristics	*N* = 46 (%)
Age, years [range]	66 [2–88]
Sex	
Female / Male	33 (72) / 12 (28)
Classification of sarcoma type	
Leiomyosarcoma	16 (35)
Malignant fibrous histiocytoma variants	16 (35)
Angiosarcoma	6 (13)
Liposarcoma	5 (11)
Rhabdomyosarcoma	3 (7)
Tumor size (cm) [range]	4.8 [1–25]
Type of surgery	
Cholecystectomy [Laparoscopy]	34 (74) [0 (0)]
Extended cholecystectomy	11 (24)
Bile duct resection	1 (2)
Unknown	1 (2)
Lymphadenectomy	
Yes / No / Unknown	4 (9) / 41 (89) / 1 (2)
Lymph node metastasis	
Yes / No / Unknown	1 (2) / 7 (15) / 38 (83)
Margin status	
R0 / R1 / R2 / Unknown	10 (22) / 0 (0) / 1 (2) / 35 (76)
Follow-up period (months) [range]	12 [0.7–324]
Recurrence	8 (17)
Liver	5 (11)
Peritoneal metastasis	3 (7)
Others (pancreas, colon)	2 (4)

Liposarcoma commonly occurs at the proximal extremities and the retroperitoneum [8]. Liposarcoma with visceral organ origin including gastrointestinal tract, gallbladder, is extremely rare with only five relevant cases reported so far [1–4]. Liposarcoma is divided into four histological subtypes: well-differentiated liposarcoma (WDL), pleomorphic liposarcoma (PL), dedifferentiated liposarcoma (DL) and myxoid liposarcoma (ML). Among these, ML is characterized as slow-growing and low-grade tumor with low metastatic potential, like well-differentiated liposarcoma [8] (Table 2).

**Table 2:** Reported cases and our case of liposarcoma with resection

Authors	Reported year	Age (years) /Sex	Preoperative diagnosis	Other lesions	Tumor size (cm)	Type of surgery	Subtypes	Lympha-denectomy	Lymph node metastasis	Recurrence	Prognosis (POM)
Hamada T	2006	49 / F	Angiosarcoma	No	25 × 23	Cholecystectomy	PL	No	No	Liver	Alive (39)
Husain EA	2009	64 / F	N/A	No	1.7	Cholecystectomy	ML	No	No	N/A	DOD (N/A)
Husain EA	2009	70 / F	N/A	No	3	Cholecystectomy	ML	No	No	N/A	DOU (N/A)
Ma Y	2014	70 / F	N/A	No	13 × 8	Cholecystectomy	ML	No	No	No recurrence	Alive (N/A)
Da Costa AC	2018	71 / F	Gallbladder cancer	No	14 × 14	Extended cholecystectomy	DL	No	No	No recurrence	Alive (8)
Our case	2020	53 / M	Liposarcoma	Left leg	4 × 3.5	Laparoscopic Cholecystectomy	ML	No	No	left popliteal fossa	Alive (28)

F, female; M, male; PL, pleomorphic liposarcoma; ML, myxoid liposarcoma; DL, dedifferentiated liposarcoma; DOD, died of disease; DOU, died of unrelated cause; POM, postoperative months.

Resection with negative margins is the standard treatment for soft-tissue sarcoma and the same principle should be applied to gallbladder liposarcoma. Unlike adenocarcinoma of gallbladder, the role of extended resection with adjacent liver and portal lymphadenectomy for gallbladder sarcoma remains unclear. Given the low risk for lymphatic spread for sarcoma in general, we did not perform portal lymphadenectomy for our patient with small gallbladder mass without sign of invasion to the liver bed.

In terms of long-term outcomes, the patients with low-grade myxoid and well-differentiated subtypes have excellent outcomes with survival rate greater than 90% at 5 years after complete resection [4, 9, 10]. Chemotherapy should be reserved for unresectable disease and adjuvant therapy after complete resection is not recommended. As our patients developed recurrence in multiple sites including left popliteal fossa at 16 months after the operation and left groin and right popliteal fossa at 19 months, he received systemic chemotherapy rather than repeat resections. Although ML is known to be more sensitive to traditional cytotoxic agents including doxorubicin or ifosfamide than other types of sarcomas [11], trabectedin was elected as his chemotherapeutic regimen based on the genomic test for his recurrent tumor. Trabectedin targets FUS-CHOP oncoprotein and shown to be effective for patients with liposarcoma with FUS-CHOP fusion gene [12].

We thought his GB sarcoma as a second primary disease based on his clinical course and treated it accordingly. However, it is not easy to prove whether this lesion was truly a second primary of multifocal disease or not, and it could be an unusual form of metastasis. In fact, multifocal sarcoma is a rare and controversial entity. ‘Multifocal’ disease judged by clinical criteria reported as rare as 1% among all types of sarcoma [13] or 4.5% among liposarcoma [14]. In two studies using genomic analysis, the tumors at different site in all patients with ‘multifocal’ myxoid liposarcoma had the monoclonality in the genomic profile and they concluded clinical ‘multifocal’ disease is likely an atypical pattern of metastatic disease [14, 15]. For our patient, unfortunately, genomic test was not available for previously resected tumor.

In conclusions, we have presented a rare case of gallbladder liposarcoma. Collaborative efforts to collect and compile pieces of information for clinicopathological features and treatment outcomes for each case is essential to establish the optimized care for this extremely rare disease.

## References

[1] Ma Y , Wei S, Peker D. An extremely rare primary gallbladder myxoid liposarcoma associated with amplification of DDIT3 gene. J Gastrointestin Liver Dis 2014;23:460–1. 25532011

[2] Husain EA , Prescott RJ, Haider SA, Al-Mahmoud RW, Zelger BG, Zelger B, et al. Gallbladder sarcoma: a clinicopathological study of seven cases from the UK and Austria with emphasis on morphological subtypes. Dig Dis Sci 2009;54:395–400. 18618258 10.1007/s10620-008-0358-z

[3] Hamada T , Yamagiwa K, Okanami Y, Fujii K, Nakamura I, Mizuno S, et al. Primary liposarcoma of gallbladder diagnosed by preoperative imagings: a case report and review of literature. World J Gastroenterol 2006;7:1472–5. 10.3748/wjg.v12.i9.1472 PMC4124333 16552824

[4] da Costa AC , Santa-Cruz F, Sena BF, Lopes A, Leite N, da Paz AR, et al. Dedifferentiated liposarcoma of the gallbladder: first reported case. World J Surg Oncol 2018;16:221. 30419915 10.1186/s12957-018-1520-5 PMC6233360

[5] Kim HH , Hur YH, Jeong EH, Park EK, Koh YS, Kim JC, et al. Carcinosarcoma of the gallbladder: report of two cases. Surg Today 2012;42:670–5. 22391981 10.1007/s00595-012-0160-6

[6] Ayoub M , Jabi R, Achraf M, Benani A, Soumia EA, Imane K, et al. Surgical management of gallbladder carcinosarcoma: a case report and review of the literature. Int J Surg Case Rep 2020;75:460–3. 33076195 10.1016/j.ijscr.2020.09.114 PMC7527620

[7] Al-Daraji WI , Makhlouf HR, Miettinen M, Montgomery EA, Goodman ZD, Marwaha JS, et al. Primary gallbladder sarcoma: a clinicopathologic study of 15 cases, heterogeneous sarcomas with poor outcome, except pediatric botryoid rhabdomyosarcoma. Am J Surg Pathol 2009;33:826–34. 19194282 10.1097/PAS.0b013e3181937bb3

[8] Lee ATJ , Thway K, Huang PH, Clinical JRL. Molecular spectrum of liposarcoma. J Clin Oncol 2018;10:151–9. 10.1200/JCO.2017.74.9598 PMC5759315 29220294

[9] Lewis JJ , Leung D, Woodruff JM, Brennan MF. Retroperitoneal soft-tissue sarcoma: analysis of 500 patients treated and followed at a single institution. Ann Surg 1998;228:355–65. 9742918 10.1097/00000658-199809000-00008 PMC1191491

[10] Linehan DC , Lewis JJ, Leung D, Brennan MF. Influence of biologic factors and anatomic site in completely resected liposarcoma. J Clin Oncol 2000;18:1637–43. 10764423 10.1200/JCO.2000.18.8.1637

[11] Jones RL , Fisher C, Al-Muderis O, Judson IR. Differential sensitivity of liposarcoma subtypes to chemotherapy. Eur J Cancer. 2005 Dec;41:2853–60. 16289617 10.1016/j.ejca.2005.07.023

[12] Assi T , Kattan J, El Rassy E, Honore C, Dumont S, Mir O, et al. A comprehensive review of the current evidence for trabectedin in advanced myxoid liposarcoma. Cancer Treat Rev 2019;72:37–44. 30468937 10.1016/j.ctrv.2018.11.003

[13] Blair SL , Lewis JJ, Leung D, Woodruff J, Brennan MF. Multifocal extremity sarcoma: an uncommon and controversial entity. Ann Surg Oncol 1998;5:37–40. 9524706 10.1007/BF02303762

[14] de Vreeze R , de Jong D, Nederlof P, Ruijter HJ, Boerrigter L, Haas R, et al. Multifocal myxoid liposarcoma--metastasis or second primary tumor? A molecular biological analysis. J Mol Diagn 2010;12:238–43. 20093386 10.2353/jmoldx.2010.090117 PMC2871731

[15] Antonescu CR , Elahi A, Healey JH, Brennan MF, Lui MY, Lewis J, et al. Monoclonality of multifocal myxoid liposarcoma: confirmation by analysis of TLS-CHOP or EWS-CHOP rearrangements. Clin Cancer Res 2000;6:2788–93. 10914725

